# Higher prevalence of viral control in HIV-1-infected women in serodiscordant relationships

**DOI:** 10.1371/journal.pone.0208401

**Published:** 2018-12-05

**Authors:** Kathryn Peebles, R. Scott McClelland, Julie Overbaugh, Barbra A. Richardson, Rose Bosire, James N. Kiarie, Carey Farquhar, Brandon L. Guthrie

**Affiliations:** 1 Department of Epidemiology, University of Washington, Seattle, Washington, United States of America; 2 Department of Medicine, University of Washington, Seattle, Washington, United States of America; 3 Department of Global Health, University of Washington, Seattle, Washington, United States of America; 4 Department of Microbiology, University of Washington, Seattle, Washington, United States of America; 5 Human Biology Division, Fred Hutchinson Cancer Research Center, Seattle, Washington, United States of America; 6 Department of Biostatistics, University of Washington, Seattle, Washington, United States of America; 7 Department of Medical Epidemiology and Biostatistics, Karolinska Institutet, Stockholm, Sweden; 8 Centre for Public Health Research, Kenya Medical Research Institute, Nairobi, Kenya; 9 Department of Obstetrics and Gynecology, University of Nairobi, Nairobi, Kenya; 10 Department of Allergy and Infectious Diseases, University of Washington, Seattle, Washington, United States of America; University of Pittsburgh, UNITED STATES

## Abstract

**Background:**

HIV-1-discordant couples that remain discordant despite repeated exposure may differ from the general population in their distribution of transmission risk factors, including low plasma viral load (PVL) in the infected partner even in the absence of antiretroviral therapy (ART).

**Methods:**

We followed two cohorts of HIV-1-infected Kenyan women: females in discordant couples (FDC) and female sex workers (FSW). We compared the distribution of undetectable (<150 copies/mL) and low PVL (<1,000 copies/mL) between the cohorts using bootstrap methods and exact Poisson regression.

**Results:**

We evaluated 296 FDC and 220 FSW. At baseline, FDC were more likely to have undetectable (RR = 6.94, bootstrap 95% CI: 3.47, 20.81) and low PVL (RR = 3.53, bootstrap 95% CI: 2.57, 5.65) than FSW. Similarly, both repeat undetectable PVL (RR = 9.36, bootstrap 95% CI: 6.04, 10.97) and repeat low (RR = 4.99, bootstrap 95% CI: 2.33, 14.04) PVL were more likely among FDC than FSW during follow-up.

**Discussion:**

We observed higher prevalence of viral control in FDC compared to FSW in the absence of ART, suggesting potentially higher prevalence of biological HIV resistance factors among discordant couples.

## Introduction

HIV-1-discordant couples, of whom one partner is HIV-positive and the other is HIV-negative, have been identified as a priority population for preventing the onward transmission of HIV, leading to expansion of treatment for people living with HIV in a discordant partnership in many countries, as well as targeted provision of pre-exposure prophylaxis to HIV-negative persons in a discordant couple. The importance of reducing HIV incidence in this population has also produced a large number of studies in which cohorts of discordant couples are enrolled to investigate risk factors for HIV transmission [1–3], evaluate the effect of interventions [4, 5], and to estimate per-act infectivity of HIV [1, 6, 7]. However, cohorts of discordant couples recruited into studies may be subject to survivor bias: discordant couples that remain discordant beyond an initial period of discordancy are more likely to have characteristics that reduce the risk of transmission. These may be behavioral factors (e.g., high condom use), host genetic factors (e.g., CCR5Δ32), or viral factors (e.g., poor replicative fitness). Indeed, such “frailty selection” has been proposed as a factor in the heterogeneity of transmission risk observed across sexual partnerships [8–10], with a correspondingly wide range of estimates of per-act infectivity of HIV [10, 11]. Among studies producing estimates of per-act infectivity of HIV, only one analysis, using data from the Rakai cohort, has included both initially concordant-HIV-negative and initially HIV-discordant couples [7]. In this analysis, estimates of per-act infectivity during the chronic phase of HIV infection were more than doubled among those couples that entered the study concordant-HIV-negative relative to those couples enrolled with discordant HIV status (risk of 0.0015 per act vs. 0.0007 per act, *p* > 0.05) [7]. Though not statistically significant, this observation lends support to a hypothesis that a longer period of discordancy is associated with lower transmission risk, suggesting that HIV-discordant couples may differ from the general population in their distribution of HIV risk factors. However, direct evidence to support this hypothesis is limited. In addition, it is unknown whether there is a biological basis contributing to reduced infectiousness of the infected partner.

One of the strongest predictors of infectivity is HIV viral load [12, 13]. Viral control in the absence of ART is rare: by one definition (>10 years of HIV infection with 90% of plasma viral load values ≤500 copies/mL and last plasma viral load value ≤50 copies/mL), only 0.15% of those infected effectively control HIV; similar definitions have yielded comparable estimates [14]. Transient control of viremia is more common, with estimates that 6.7% of patients have two consecutive viral load values below 500 copies/mL [15]. Such viral control may contribute to longer duration of serodiscordancy among discordant couples.

We hypothesized that viral control is more prevalent among infected partners in HIV-discordant couples compared to HIV-infected individuals who are not in a stable discordant relationship. To test this hypothesis, we compared plasma viral load in two cohorts of HIV-infected women who previously participated in research conducted by our group in which the same viral load assay was used. These cohorts included women in HIV-discordant couples and women engaged in transactional sex.

## Methods

### Study participants

Study participants were drawn from two cohorts of HIV-infected women, described in detail below. To maximize exchangeability of the two groups of women with respect to measurement of viral control, we limited the follow-up period for each woman to one year and a maximum of two viral load measurements. We excluded all viral load measurements following ART initiation.

### Cohort 1: Females in HIV-1-discordant couples (FDC)

HIV-1-discordant couples were recruited from voluntary counseling and testing (VCT) centers in Nairobi, Kenya from September 2007 to December 2009. Participants consented to 2 years of follow-up with quarterly study visits as part of a study of HIV-specific cellular immune responses and HIV transmission. Eligible couples reported sex ≥3 times in the 3 months prior to screening and planned to remain together for the duration of the study. Women could not be pregnant at enrollment and HIV-infected participants could not have a history of clinical AIDS (WHO stage IV) and were not currently on ART.

At each quarterly follow-up visit, participants were asked if they had started ART since their last study visit. Additionally, at the end of the study, participants were asked if they had started ART during the study period, and if so, when they had started therapy. Any visits following the earliest reported date of ART initiation were excluded from this analysis.

The Kenyatta National Hospital Ethics and Research Committee at the Kenyatta National Hospital and the University of Washington Institutional Review Board approved the study. All participants provided written informed consent.

### Cohort 2: Female sex workers (FSW)

An ongoing open cohort study of women engaging in transactional sex and thus at high risk for HIV acquisition was initiated in February 1993 [16]. Most women engaged in transactional sex to supplement income earned at bars and nightclubs, with approximately one-quarter of women reporting more than one sexual partner [17, 18]. Detailed procedures for enrollment and follow-up of HIV-1-seropositive women in the cohort have been described previously [18]. Briefly, women who acquired HIV during follow-up in the high-risk HIV-seronegative cohort were identified beginning in 1993. In addition, women who were HIV-1-seropositive at initial screening were offered enrollment beginning in 2001. Blood was collected every 3 months and processed as described [19]. ART was provided to eligible participants according to the Kenyan National Guidelines beginning in 2004. Any visits following the earliest reported date of ART initiation were excluded from this analysis.

The Kenyatta National Hospital Ethics and Research Committee and the University of Washington Institutional Review Board approved the study. All participants provided written informed consent.

### Laboratory methods

HIV-1 RNA levels were measured in plasma from blood samples collected using the Hologic/Gen-Probe HIV-1 viral load assay (Hologic Corporation, San Diego, CA, USA). CD4 cell counts for FDC were measured on blood samples collected at enrollment and every 6 months during follow-up using a FACSCalibur flow cytometer (BD Bioscience, Franklin Lakes, NJ). For FSW, CD4 cell counts were measured every three months beginning in 1998, using a manual system (Cytosphere, Coulter, Hialeah, FL) until 2004, and FACSCount flow cytometer (BD Bioscience, Franklin Lakes, NJ) thereafter.

### Assessment of viral control

Viral load measurements varied in the analytic lower limit of detection across sets of viral load assays. However, the lower limit of detection was consistently less than 150 viral copies/mL. Thus, we defined a universal threshold of 150 viral copies/mL. We defined low viral load as <1,000 viral copies/mL, following the current WHO definition of virologic failure [20]. We assessed the sensitivity of the threshold used to define low viral load by repeating analyses with the additional definition of viral load <2,000 copies/mL, as this is a commonly used threshold used to define viral control [21, 22]. We aimed to compare viral control among women with chronic HIV infection, and thus excluded viral load measurements taken within one year of seroconversion and women with a history of clinical AIDS at baseline.

### Statistical methods

Our primary analysis compared baseline and durable viral control between the two cohorts. Given that the study of discordant couples enrolled HIV-positive individuals with infection of unknown duration, while seroconversion occurred on-study for FSW, a comparison of viral load using a measure of viral load from shortly after seroconversion for FSW and a measure of viral load from an unknown point of chronic infection for FDC may be systematically biased. We therefore sought to increase comparability between the cohorts with respect to time-from-infection by conducting bootstrap analyses on 5,000 datasets in which we randomly selected a “baseline” visit for each FSW participant from among all follow-up visits >1 year after seroconversion during which she had no history of clinical AIDS (an exclusion criteria for the FDC cohort) and was not on ART, as well as a single visit one year (within a window of 30 days prior to and 90 days after the one-year date) subsequent to the “baseline”. We defined durable viral control as viral control at baseline and at a single follow-up visit one year following the baseline visit (within a window of 30 days prior to and 90 days after the one-year date).

We estimated the relative risk of viral control among FDC relative to FSW with exact Poisson regression for each bootstrapped dataset, and report the median relative risk and 95% confidence interval from the distribution of relative risks obtained from the 5,000 bootstrapped datasets. The number of FSW achieving each outcome of interest is shown as the mean obtained across the 5,000 bootstrapped datasets. We set critical alpha to 0.05 and performed two-tailed tests. We used Stata version 14.1 and R version 3.3.1 for all analyses.

### Sensitivity analyses

Enrollment in the study of discordant couples required that the HIV-positive individuals be ART-naïve. Given the benefits conferred by study participation, including enhanced clinical care and a small monetary reimbursement of 300 shillings (approximately 4 USD), participants may have been incentivized to misreport previous ART exposure; any such incentive would no longer be present once enrolled. We therefore conducted sensitivity analyses in which we excluded the data from eight women in the FDC cohort with viral control at baseline who subsequently reported ART initiation during study follow-up.

Herpes simplex virus type 2 (HSV-2) tests conducted in initial years of cohort establishment among FSW demonstrated almost universal HSV-2 infection. HSV-2 testing was therefore discontinued in subsequent years, precluding a comparison of the distribution of HSV-2 across the two cohorts of FDC and FSW. To account for possible confounding by differences in HSV-2 status between the cohorts, we conducted a sensitivity analysis in which we assumed all FSW were positive for HSV-2 (following test results in early years) and excluded all FDC who were HSV-2 negative.

Finally, we also conducted quantitative bias analysis to evaluate the extent of bias induced by an unmeasured confounder that would be necessary to nullify our estimates [23]. This bias analysis estimates a relative risk adjusted for an unmeasured confounder, given the observed distribution of the outcome of viral control among FDC and FSW, hypothetical distributions of the unmeasured confounder among FDC and FSW, and the hypothetical strength of association between the confounder and outcome. Adjusted relative risk values close to one thereby identify the combination of the magnitude of differential distribution of the unmeasured confounder among FDC and FSW and the strength of association between the unmeasured confounder and the outcome necessary to explain the observed, unadjusted relative risks.

## Results

### Participant characteristics

Study participants were 296 HIV-positive FDC and 220 HIV-positive FSW women. The mean age at enrollment among FDC women was 29.3 (SD 6.7) compared to a mean age of 34.1 (SD 6.6) among FSW (Table 1). More FSW (100, 45.5%) had low education, defined as fewer than 8 years of education, compared to FDC (71, 24.0%). Mean age at first sex was similar in the two cohorts (FDC: 17.7, SD 2.6; FSW: 16.7, SD 2.4). Baseline CD4 counts were lower among FSW (median 406 cells/mm^3^, IQR 260–580) than among DC women (median 477 cells/mm^3^, IQR 311–677), while viral load was slightly higher among FSW (4.79 log_10_ copies/mL, IQR 4.21–5.37) than among FDC women (median 4.59 log_10_ copies/mL, IQR 3.77–5.20). Most (71.6%) DC women had a follow-up visit, compared to 32.8% of FSW with a follow-up within a window of 30 days prior to and 90 days after one year from baseline.

**Table 1:** Baseline characteristics of study participants.

	FDC		FSW	
	*N*		*N*	
Age, mean (SD)	296	29.3 (6.7)	220	34.1 (6.6) ^a^
Low education, *n* (%)	296	71 (24.0)	220	100 (45.5)
Age at first sex, mean (SD)	296	17.7 (2.6)	216	16.7 (2.4)
CD4 at baseline, median (IQR)	277	477 (311–677)	172	406 (260–578) ^a^
Year of enrollment, median (IQR)	296	2008 (2008–2009)	220	2002 (1999–2005) ^a^
Viral load, median (IQR)	296	4.59 (3.77–5.20)	220	4.79 (4.21–5.37) ^a^

FDC = females in discordant couples; FSW = female sex workers

^a^ Values are means of 5,000 bootstrapped datasets.

Viral load measurements taken from two years of follow-up among FDC indicate trajectories consistent with chronic HIV infection (S1 Fig). Similarly, the distribution across the 5,000 bootstrapped datasets of time since seroconversion at the baseline visit of FSW is indicative of chronic HIV infection (S2 Fig).

### Viral control

**Fig 1:**
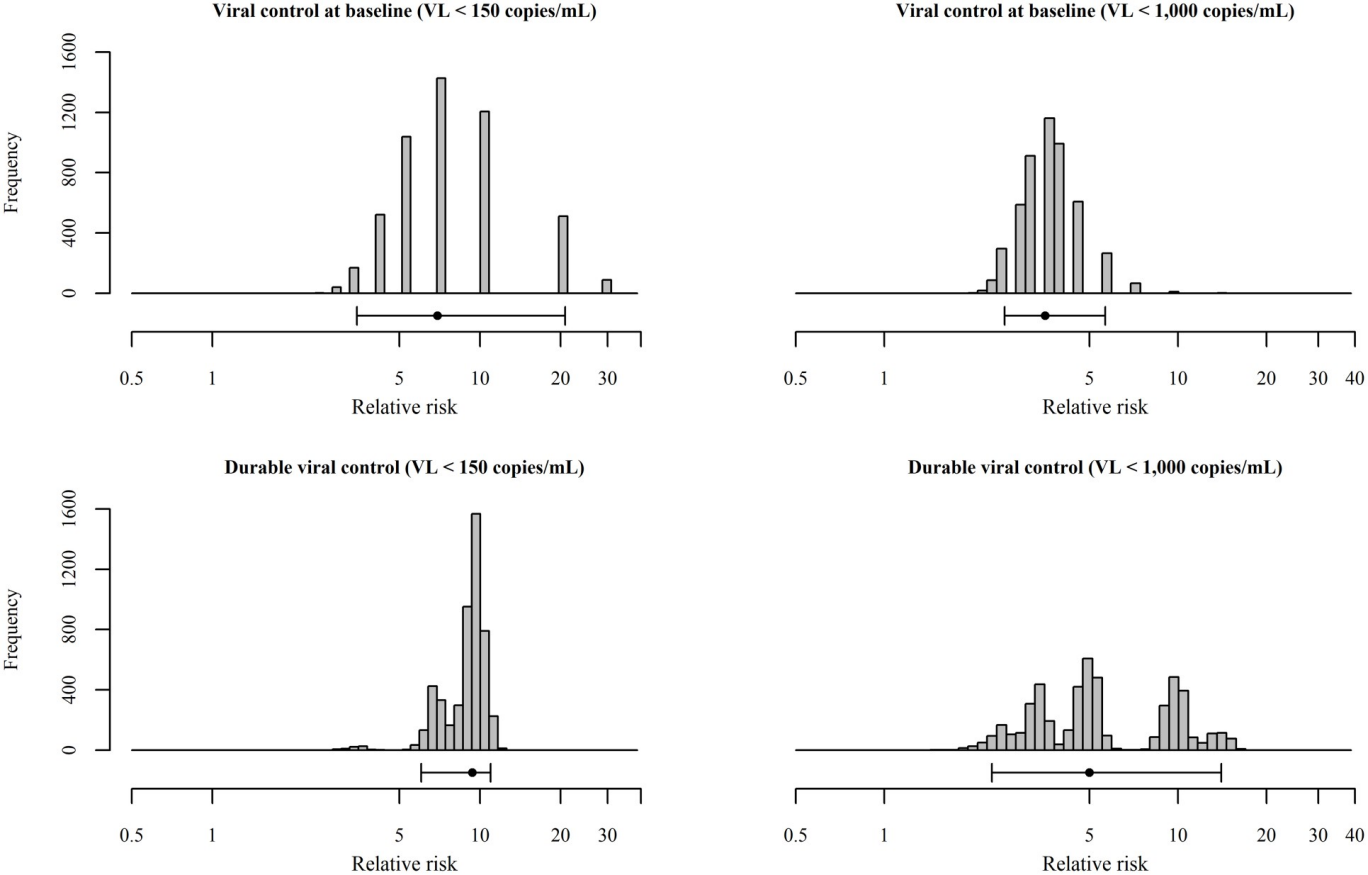
Distribution of relative risk of viral control estimates from 5,000 bootstrapped datasets. Baseline for FSW is defined as a randomly selected visit from among each woman’s total follow-up visits. The median and 95% confidence interval of the bootstrap distribution is displayed below the distribution.

Twenty-eight (9.5%) FDC women and 3.1 (1.4%) FSW (RR = 6.94, bootstrap 95% CI: 3.47, 20.81) had undetectable plasma viral load (PVL) at baseline (Table 2). Among those with initially undetectable PVL and a one-year follow-up visit, repeated undetectable PVL occurred in 80.0% (20/25) of initially undetectable FDC and 7.7% (0.24/3.1) of initially undetectable FSW. Low PVL, defined as viral load less than 1,000 copies/mL, at baseline was observed in 38 (12.8%) FDC and 8.0 (3.6%) FSW (RR = 1.26, bootstrap 95% CI: 0.94, 1.73). Among those with low PVL at baseline and a one-year follow-up visit, 93.6% (29/31) FDC and 25.0% (2.0/8.0) FSW had a subsequent low PVL measurement. Among all women with a one-year follow-up visit, 20 (9.4%) FDC had undetectable viral load at baseline and one year later, compared to 0.2 (0.3%) FSW (RR = 9.36, bootstrap 95% CI: 6.04, 10.97; Fig 1, Table 2). Twenty-nine (13.7%) FDC and 2.0 (2.8%) FSW had low PVL at both baseline and at a one-year follow-up visit (RR = 4.99, bootstrap 95% CI: 2.33, 14.04; Fig 1, Table 2). Results for analyses of low viral load with the definition of fewer than 2,000 copies/mL produced comparable results to those obtained when defining low viral load as fewer than 1,000 copies/mL (Table 2).

**Table 2:** Low and undetectable viral load among women in a discordant partnership relative to women engaged in transactional sex.

	*N*	FDC *n* (%)	*N* ^a^	FSW *n* (%) ^a^	Relative risk (95% confidence interval) ^b^	*p*
At baseline visit						
Viral load < 150 copies/mL	296	28 (9.5)	220	3.1 (1.4)	6.94 (3.47, 20.81)	< 0.001
Viral load < 1,000 copies/mL	296	38 (12.8)	220	8.0 (3.6)	3.53 (2.57, 5.65)	< 0.001
Viral load < 2,000 copies/mL	296	48 (16.2)	220	12.2 (5.5)	2.97 (2.23, 3.96)	< 0.001
Durable viral control ^c^						
Viral load < 150 copies/mL	212	20 (9.4)	72.2	0.2 (0.3)	9.36 (6.04, 10.97)	< 0.001
Viral load < 1,000 copies/mL	212	29 (13.7)	72.2	2.0 (2.8)	4.99 (2.33, 14.04)	0.001
Viral load < 2,000 copies/mL	212	33 (15.6)	72.2	2.8 (3.9)	3.89 (2.05, 13.11)	< 0.001

^a^ Values are means of 5,000 bootstrapped datasets.

^b^ Values are median and 2.5^th^ and 97.5^th^ percentiles of distribution of 5,000 bootstrapped datasets.

^c^ Defined as viral control at baseline and one follow-up visit. Calculations include all women.

FDC = females in discordant couples; FSW = female sex workers

### Sensitivity analyses

Eight FDC with viral control at baseline subsequently reported ART initiation during study follow-up. The pattern of CD4 counts and self-reported ART for 1 woman (ID 213) is suggestive of ART initiation prior to baseline and a misreport of baseline ART exposure, while CD4 count and self-reported ART use patterns for the remaining 7 women do not suggest baseline misreporting of ART (S1 Table). Exclusion of all 8 FDC with viral control at baseline and subsequent initiation of ART did not qualitatively change the point estimates nor bootstrap confidence intervals obtained in analyses of VL differences between FDC and FSW (S2 Table).

A total of 103 (34.8%) FDC were HSV-2-negative at enrollment and were excluded in a sensitivity analysis to assess the potential impact of confounding by HSV-2 status. In this analysis, in which all FDC were HSV-2-positive and all FSW were assumed HSV-2-positive, estimates of the relative risk were similar to those in our primary analysis (S3 Table).

In quantitative bias analyses, unmeasured confounding could nullify our unadjusted relative risk estimates only with extreme combinations of a confounder-outcome relationship and disparate distributions of the confounder between FDC and FSW (S3 Fig). For example, with a threshold of < 150 copies/mL, our baseline estimate of relative risk could be nullified if the relative risk between the confounder and the outcome were as strong as 6 and the absolute difference in the prevalence of the confounder between FDC and FSW was 81% (S3 Fig).

## Discussion

We observed higher prevalence of viral control in women in discordant couples compared to women engaged in transactional sex in the absence of ART. This result was consistent for both baseline and durable viral control, and was robust to sensitivity analyses in which a portion of DC women were excluded from the sample due to possible unreported ART use or HSV-2 seronegative status. This higher prevalence of viral control among FDC lends support to the hypothesis that discordant couples who have remained discordant for some duration may differ from the general population of HIV-infected and HIV-susceptible individuals in important ways related to HIV transmission risk. Thus, this population is likely enriched in both behavioral and biological resistance factors. Given that highly effective and acceptable preventive therapies are now widely available, such as treatment-as-prevention and pre-exposure prophylaxis, it is infeasible to enroll new observational cohorts of discordant couples to evaluate these factors. However, archived samples from the many historical cohorts of discordant couples may provide new insights to biological factors associated with lower transmission risk, potentially pointing to new preventive therapies.

In this study, we show that discordant couple populations are likely to be less infectious overall due to a higher fraction of individuals who control VL in the absence of ART. Such differences would suggest caution when trying to generalize estimates of per-act infectivity obtained from among cohorts of discordant couples, as these estimates may be underestimates of per-act infectivity in the general population. Use of these estimates in mathematical models of the general population may be more robust if estimates of per-act infectivity that account for viral load are used, such as the estimates provided in Hughes, et al. [1]. However, given that other co-factors also affect transmission rates and have not yet been included in statistical models of per-act HIV transmission probability, mathematical models may also consider treating per-act infectivity as an uncertainty parameter with upper bounds greater than reported estimates of per-act infectivity.

Much of the existing literature regarding viral control has focused on elite controllers. However, given the rarity with which transmission occurs at low viral load levels [13], investigations of viral control defined with relatively higher thresholds are relevant for identifying factors associated with reduced risk of transmission. In a cohort of HIV-positive United States military personnel, 3.9% of study participants exhibited viral control, defined as having any three viral load measures below 2,000 copies/mL over the course of one or more years of follow-up [24], while in a European seroconverter cohort, 6.7% of individuals were classified to have durable viral control, defined as any two consecutive viral load measures < 500 copies/mL from among two or more follow-up visits [15]. While the variation in definitions precludes a direct comparison of viral control prevalence estimates across these studies, these estimates are similar to the prevalence of viral control we observed among FSW and lower than what we observed among FDC (3.9% and 15.6%, respectively, with viral control defined as two viral load measures <2,000 copies/mL).

Our study benefitted from a comparison of demographically similar cohorts, though we cannot rule out that our estimates are confounded by unmeasured variables. In particular, the distribution of sexually transmitted infections (STI) likely differed between the cohorts. While many STI are known to affect genital tract HIV viral load [25], only HSV-2 is an established predictor of plasma viral load [26]. We therefore conducted a sensitivity analysis in which we sought to compare only the subset of FDC who tested positive for HSV-2 and the full cohort of FSW, all of whom we assumed to be HSV-2 positive given results from tests conducted in the initial years of cohort establishment. This sensitivity analysis produced similar relative risk estimates as in our primary analysis. Furthermore, quantitative bias analyses demonstrated that only extreme combinations of bias parameters could nullify our unadjusted relative risk estimates. Additionally, previous analyses in this cohort of FSW have found viral set point and viral load trajectories in this cohort to be comparable to those in general populations [19], suggesting that additional confounders with respect to viral load are not present in this cohort of women who engage in transactional sex. Nonetheless, future research may benefit from more complete ascertainment of potential confounders such as HSV-2 status, as well as contemporaneous measures of viral load from HIV-positive individuals in discordant relationships and HIV-1-positive individuals from the general population.

Unreported antiretroviral use by HIV-1-infected women in discordant couples at study enrollment may result in an overestimate of naturally occurring viral control. As women in the cohort of FSW received ART as part of study participation, concerns that ART use is misclassified are primarily limited to the cohort of FDC. While the absence of drug concentration measures precludes confirmation of antiretroviral use in FDC participants, negligible levels of community knowledge of the efficacy of treatment for prevention at the time of the study would likely result in similar rates of treatment seeking among discordant couples as in other groups of individuals living with HIV. Additionally, sensitivity analyses in which we excluded women with viral control at baseline who subsequently reported ART initiation during study follow-up did not produce results that differed qualitatively from analyses in which these women were included.

We defined viral control on the basis of plasma viral load due to greater availability of data for this measure. However, vaginal viral load is the more relevant measure for transmission potential. Measures of vaginal viral load were available for FDC and a subset of FSW, and showed moderate correlation with plasma viral load (*r* = 0.66 and *r* = 0.47, respectively), indicating that plasma viral load is an acceptable proxy for vaginal viral load. Use of plasma viral load is also consistent with its use as a predictor of transmission in other studies. [1, 13]

In conclusion, we observed higher prevalence of viral control among ART-naïve HIV-positive women in discordant couples than among HIV-positive women engaged in transactional sex. The results from this study highlight serodiscordant couples as a potentially important population for investigations of why some individuals naturally suppress virus, as well as potential identification of additional biological factors associated with lower risk of HIV transmission and acquisition.

## Supporting information

S1 Fig Viral load trajectory among FDC over two years of follow-up. The width of each line at a given time point is proportional to the number of women contributing a viral load measure at that time point. Measures of viral load from women on ART are excluded.
(TIF)

S2 Fig Distribution across 5,000 bootstrapped datasets of the time since seroconversion to a randomly selected baseline visit among 220 women engaged in transactional sex. All visits following a clinical diagnosis of AIDS are excluded from analyses.
(TIF)

S3 Fig Quantitative bias analysis plots. Relative risk values adjusted for an unmeasured confounder are shown for given observed distribution of viral control, hypothetical distribution of an unmeasured confounder among FDC and FSW, and hypothetical strength of relationship between the unmeasured confounder and viral control. Adjusted relative risks between 0.75 and 1.33, indicating approximate nullification of the observed, unadjusted association, are shown in black. Adjusted relative risks less than 0.1 or greater than 10 are excluded from plots. a) Baseline viral control, threshold of < 150 copies/mL; b) Baseline viral control, threshold of < 1,000 copies/mL; c) Baseline viral control, threshold of < 2,000 copies/mL; d) Durable viral control, threshold of < 150 copies/mL; e) Durable viral control, threshold of < 1,000 copies/mL; f) Durable viral control, threshold of < 2,000 copies/mL.
(PDF)

S1 Table Self-reported ART status, viral load, and CD4 count among women excluded in sensitivity analyses. (DOCX)

S2 Table Sensitivity analysis in which women in discordant couples who are HSV-2-negative are excluded. (DOCX)

S3 Table Sensitivity analysis in which women in discordant couples with low or non-detectable viral load at baseline who report ART at any subsequent follow-up visit are excluded. (DOCX)
