# Supplementary figures and images for: Higher prevalence of viral control in HIV-1-infected women in serodiscordant relationships
Source: PLoS One. 2018 Dec 5;13(12):e0208401. doi: 10.1371/journal.pone.0208401 (PMC6281234; doi:10.1371/journal.pone.0208401)

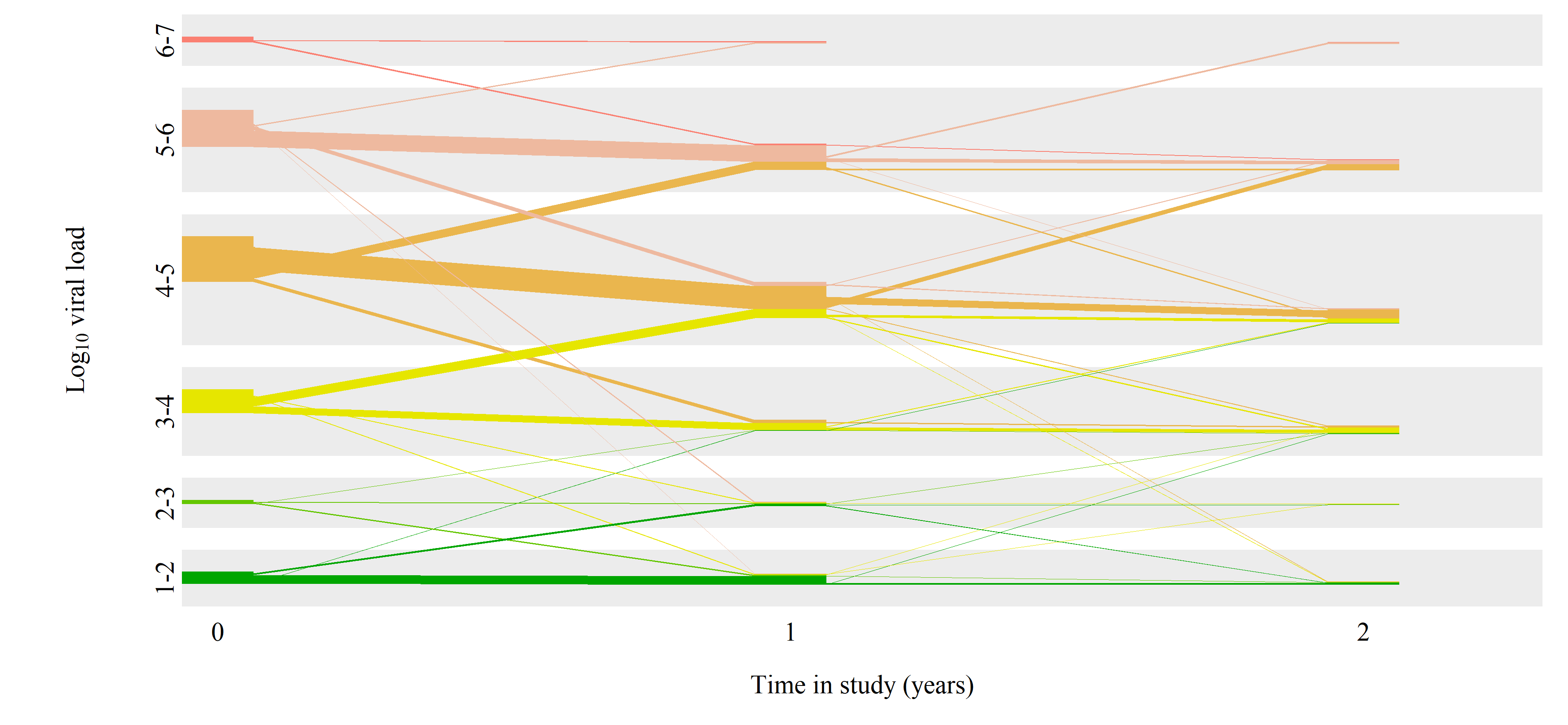

Supplement: S1 Fig — The width of each line at a given time point is proportional to the number of women contributing a viral load measure at that time point. Measures of viral load from women on ART are excluded. (TIF) [file pone.0208401.s001.tif]

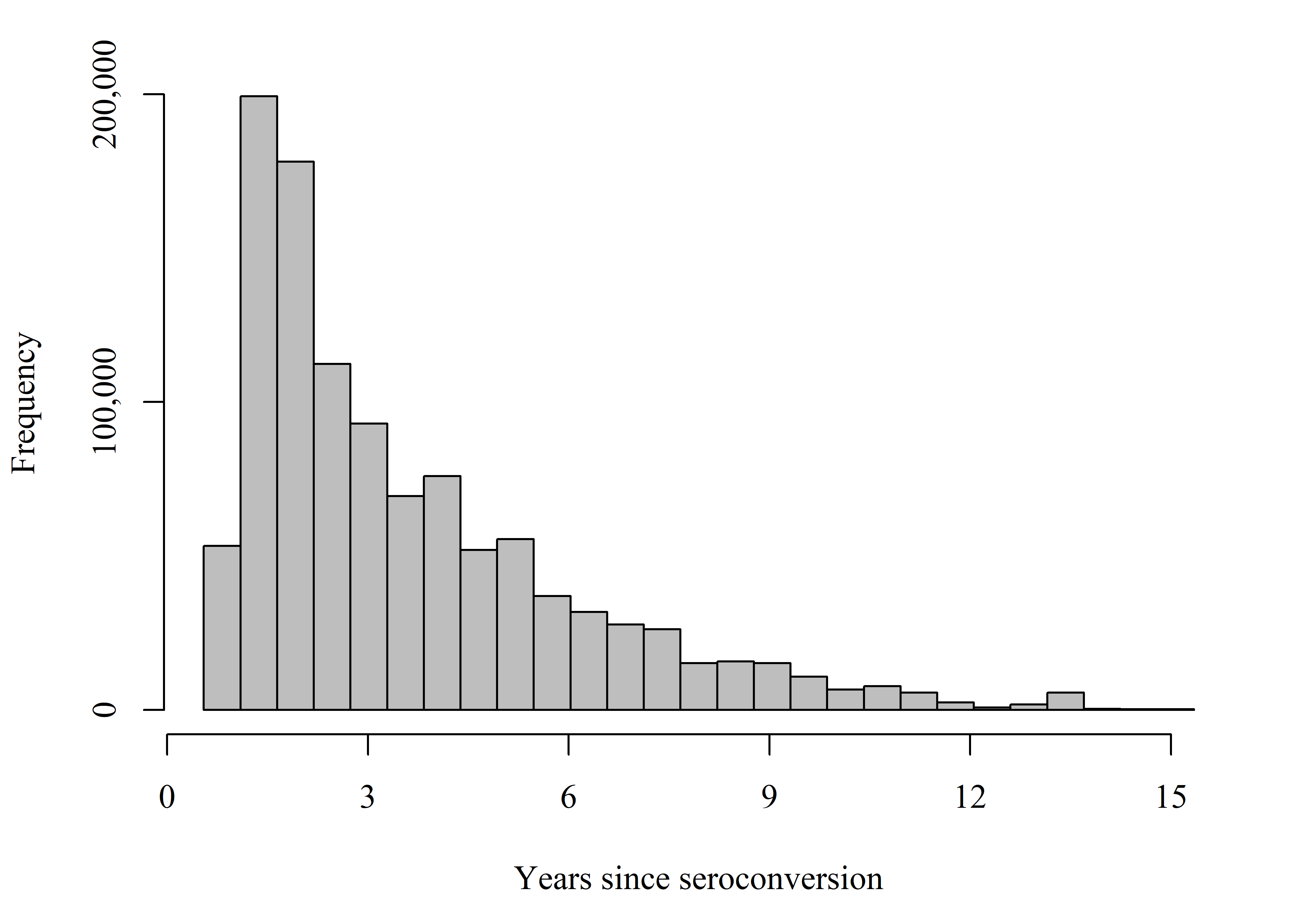

Supplement: S2 Fig — All visits following a clinical diagnosis of AIDS are excluded from analyses. (TIF) [file pone.0208401.s002.tif]

**A**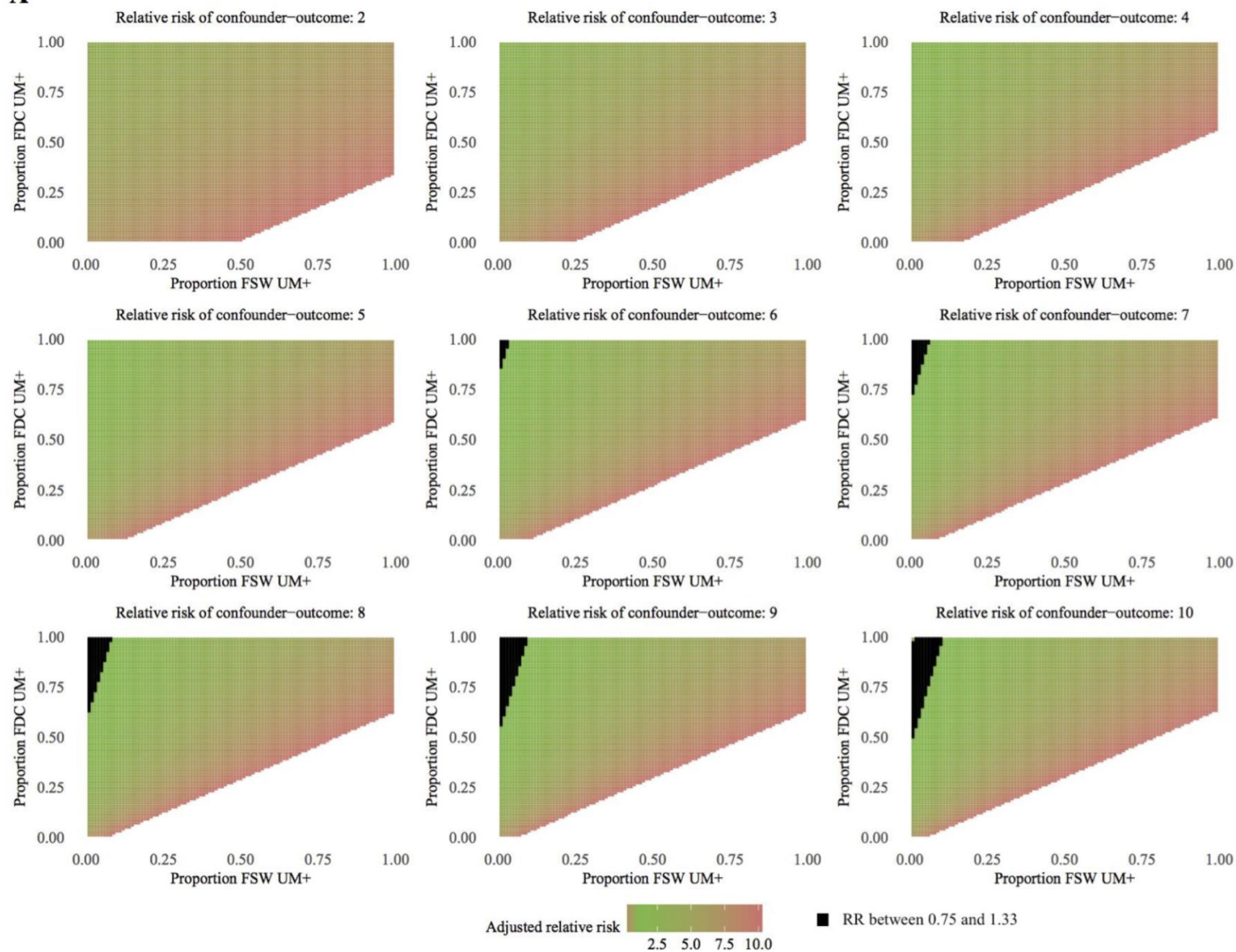

**B**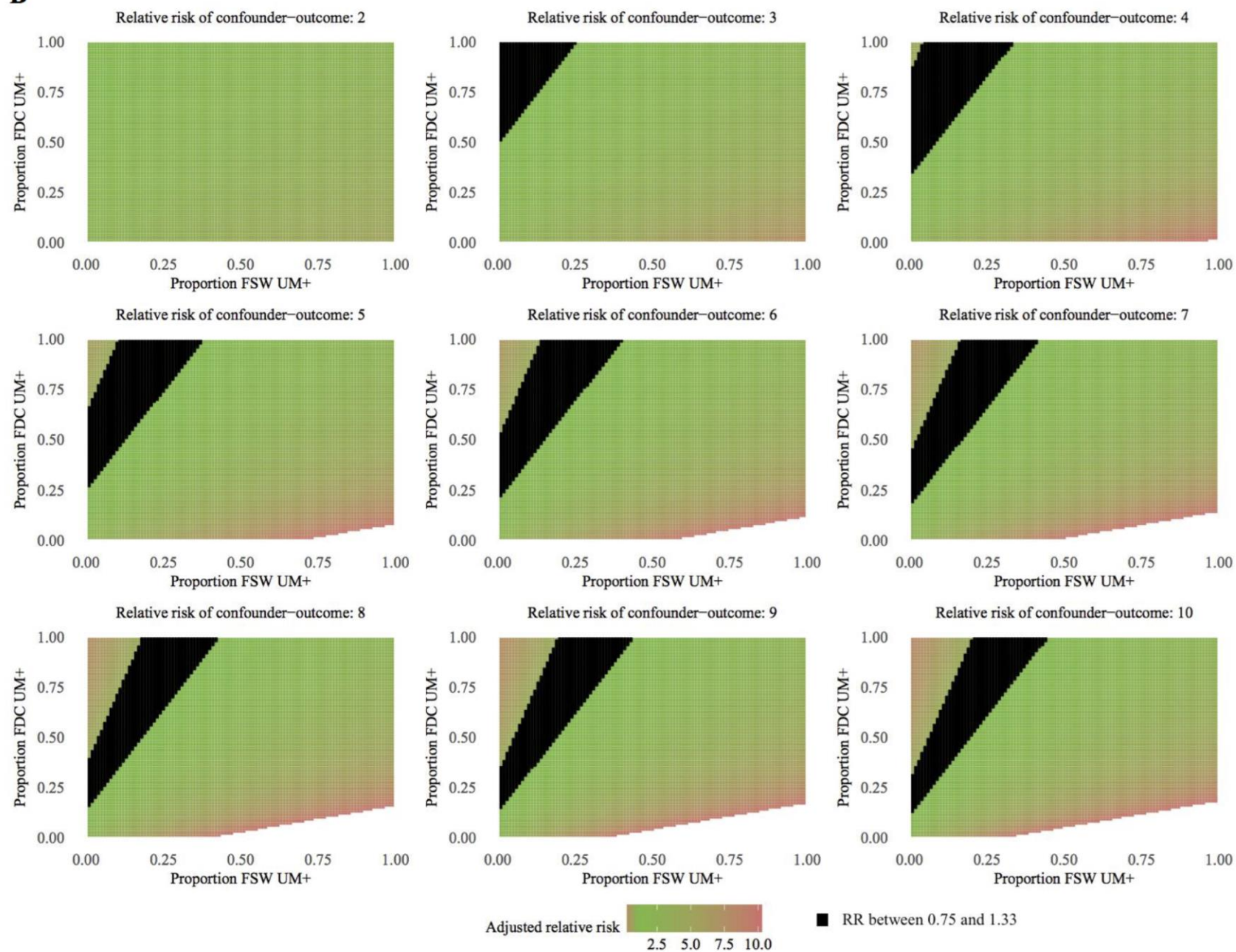

C

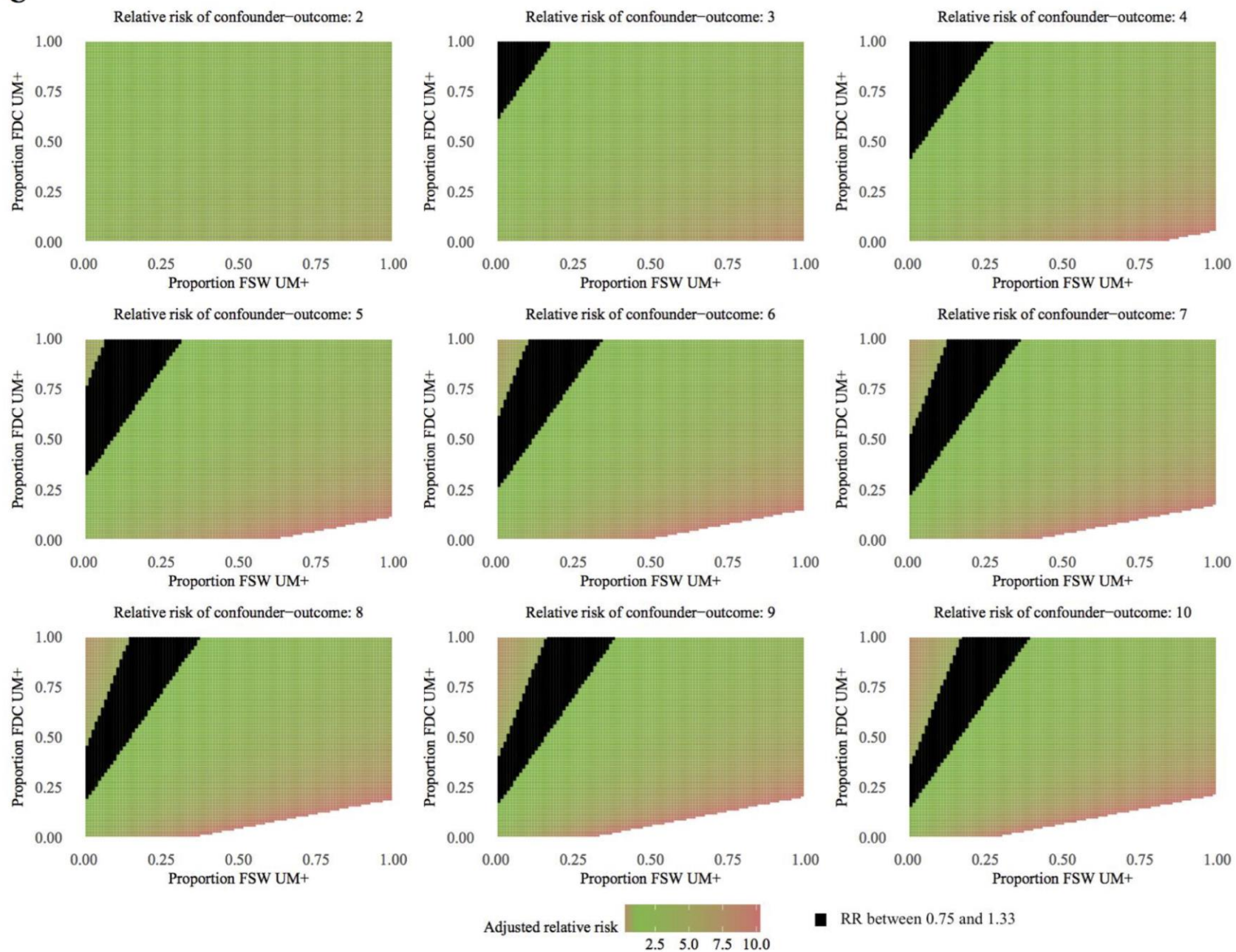

**D**

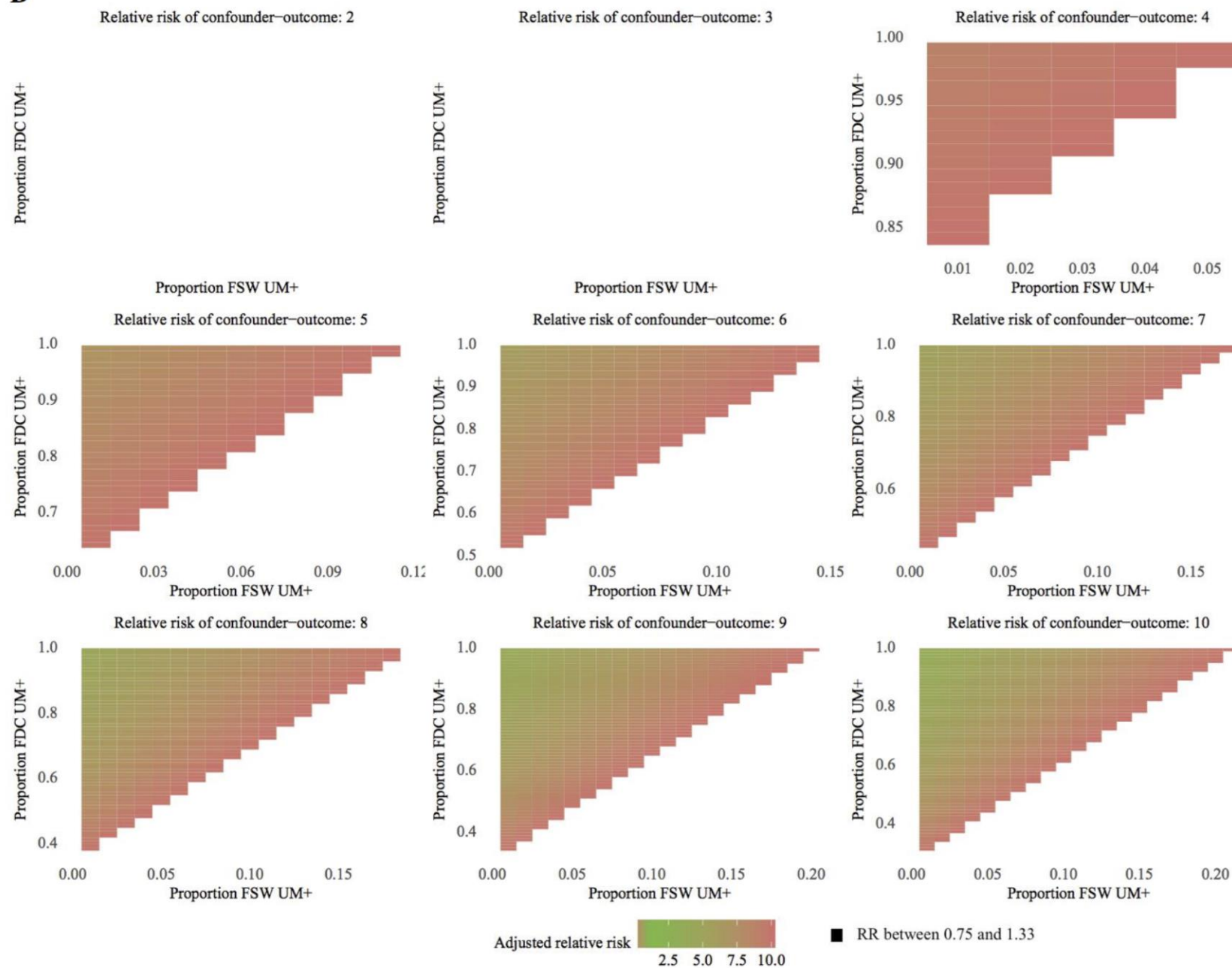

**E**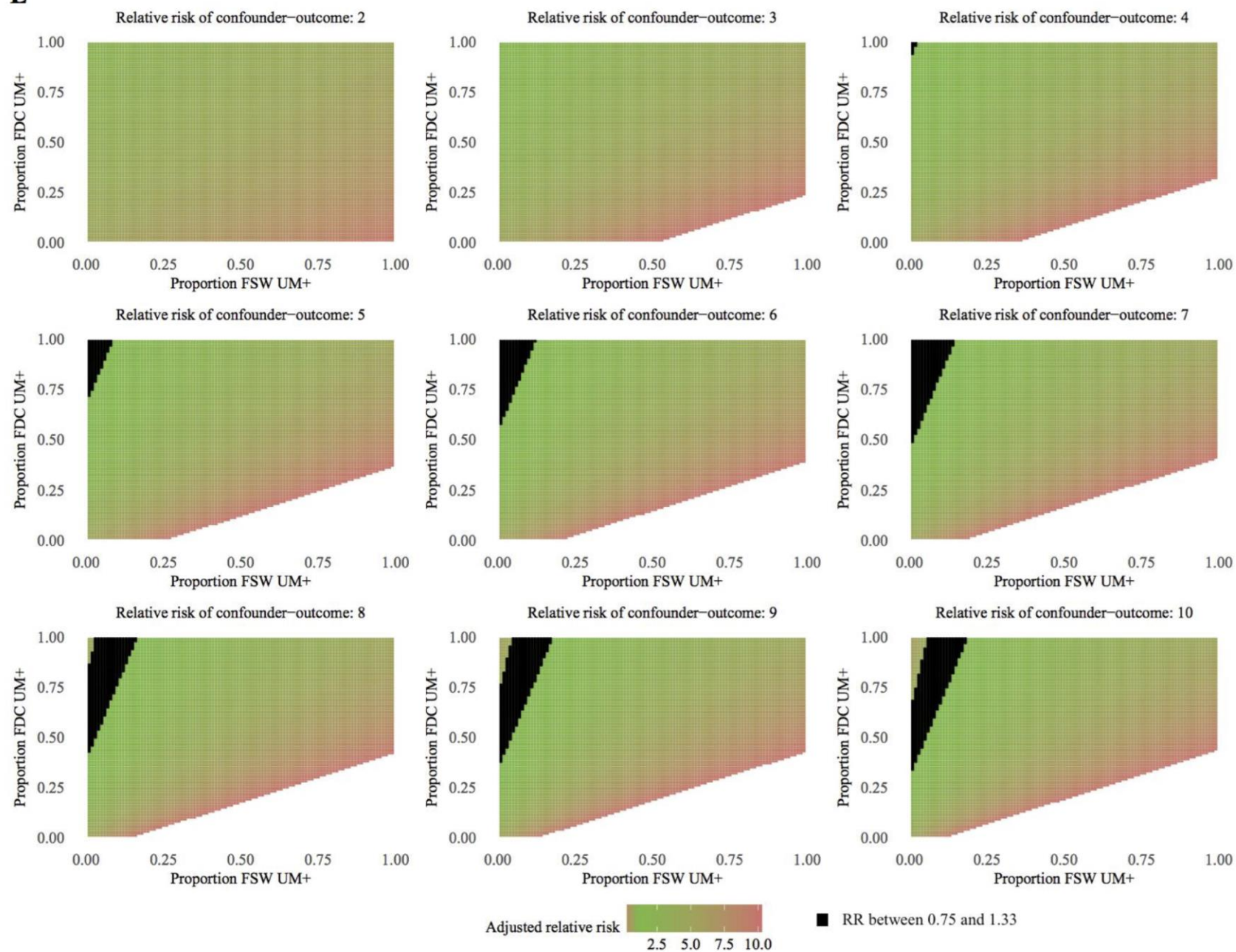

**F**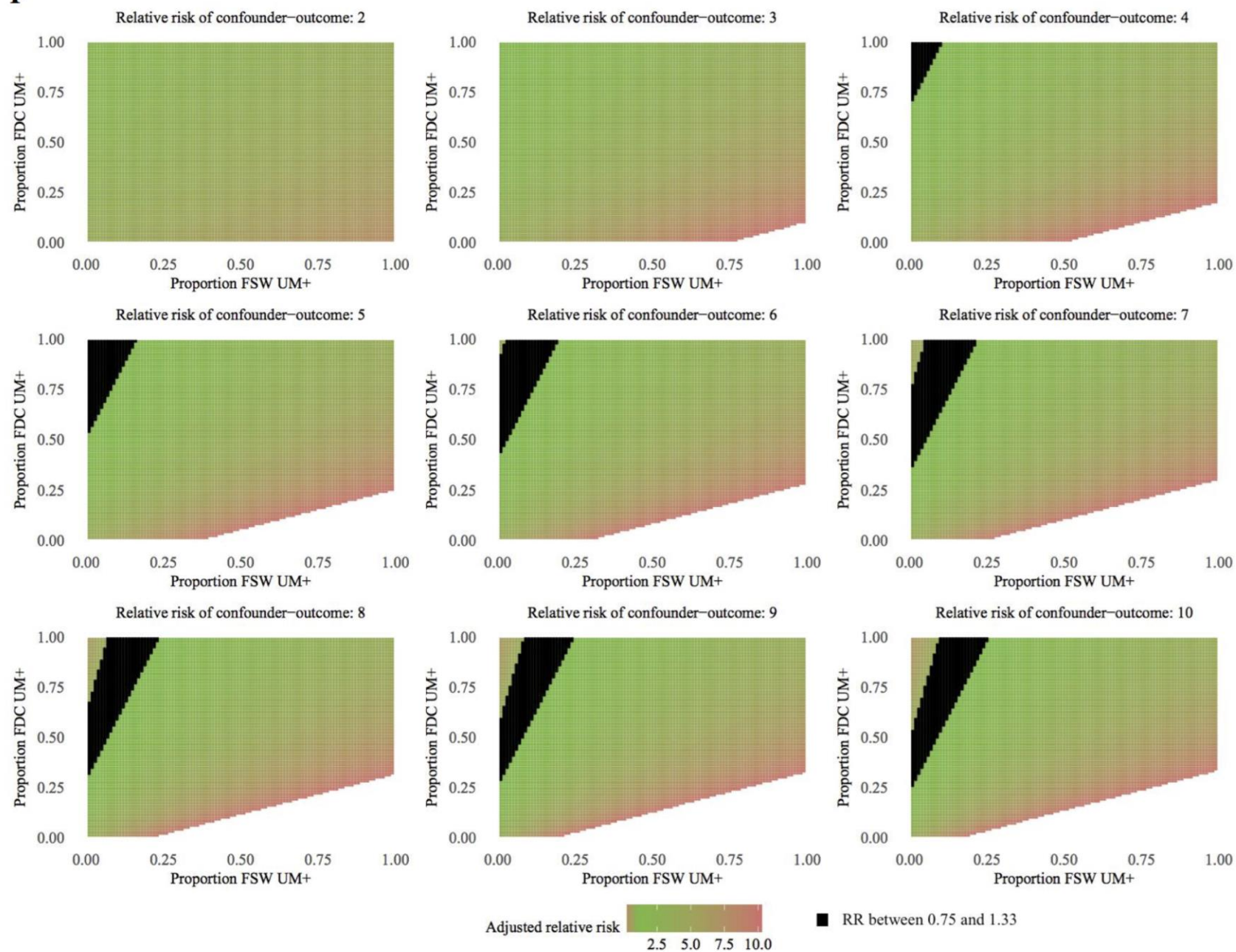

Supplement: S3 Fig — Relative risk values adjusted for an unmeasured confounder are shown for given observed distribution of viral control, hypothetical distribution of an unmeasured confounder among FDC and FSW, and hypothetical strength of relationship between the unmeasured confounder and viral control. Adjusted relative risks between 0.75 and 1.33, indicating approximate nullification of the observed, unadjusted association, are shown in black. Adjusted relative risks less than 0.1 or greater than 10 are excluded from plots. a) Baseline viral control, threshold of < 150 copies/mL; b) Baseline viral control, threshold of < 1,000 copies/mL; c) Baseline viral control, threshold of < 2,000 copies/mL; d) Durable viral control, threshold of < 150 copies/mL; e) Durable viral control, threshold of < 1,000 copies/mL; f) Durable viral control, threshold of < 2,000 copies/mL. (PDF) [file pone.0208401.s003.pdf]
