# Supplementary material for: Higher prevalence of viral control in HIV-1-infected women in serodiscordant relationships
Source: PLoS One. 2018 Dec 5;13(12):e0208401. doi: 10.1371/journal.pone.0208401 (PMC6281234; doi:10.1371/journal.pone.0208401)
Supplement: S1 Table — (DOCX) [file pone.0208401.s004.docx]

| Participant |  | Time point | | | | | | | | |
| --- | --- | --- | --- | --- | --- | --- | --- | --- | --- | --- |
|  |  | Baseline | 3 months | 6 months | 9 months | 12 months | 15 months | 18 months | 21 months | 24 months |
| 014 | ART use * | No | No | No | No | No | No | No | No | No |
|  | Viral load | ND | ^†^ | ^†^ | ^†^ | ND | ^†^ | ^†^ | ^†^ | ND |
|  | CD4 count | 269 | ^†^ | 253 | ^†^ | 194 | ^†^ | 416 | ^†^ | 262 |
| 213 | ART use | No | Yes | Yes | Yes | Yes | Yes | Yes | Yes | Yes |
|  | Viral load | ND | ^†^ | ^†^ | ^†^ | ND | ^†^ | ^†^ | ^†^ | ND |
|  | CD4 count | 487 |  | 595 |  | 529 |  | 732 |  | 725 |
| 411 | ART use | No | No | No | No | No | No | No | No | Yes |
|  | Viral load | ND | ^†^ | ^†^ | ^†^ | ND | ^†^ | ^†^ | ^†^ | ND |
|  | CD4 count | 311 | ^†^ | 345 | ^†^ | 348 | ^†^ | 443 | ^†^ | 418 |
| 514 | ART use * | No | No | No | No | No | No | No | No | No |
|  | Viral load | ND | ^†^ | ^†^ | ^†^ | ND | ^†^ | ^†^ | ^†^ | ND |
|  | CD4 count | 282 | ^†^ | 554 | ^†^ | 148 | ^†^ | 533 | ^†^ | 516 |
| 712 | ART use | No | Yes | Yes | Yes | Yes | Yes | ^m^ | ^m^ | ^m^ |
|  | Viral load | ND | ^†^ | ^†^ | ^†^ | 1,155 | ^†^ | ^†^ | ^†^ | ^m^ |
|  | CD4 count | 106 | ^†^ | 175 | ^†^ | 259 | ^†^ | ^m^ | ^†^ | ^m^ |
| 814 | ART use | No | No | No | No | Yes | ^m^ | Yes | ^m^ | ^m^ |
|  | Viral load | ND | ^†^ | ^†^ | ^†^ | ND | ^†^ | ^†^ | ^†^ | ^m^ |
|  | CD4 count | 190 | ^†^ | 181 | ^†^ | 263 | ^†^ | 162 | ^†^ | ^m^ |
| 811 | ART use * | No | No | No | No | No | No | ^m^ | ^m^ | ^m^ |
|  | Viral load | ND | ^†^ | ^†^ | ^†^ | ND | ^†^ | ^†^ | ^†^ | ^m^ |
|  | CD4 count | 390 | ^†^ | 319 | ^†^ | 301 | ^†^ | ^m^ | ^†^ | ^m^ |
| 416 | ART use | No | No | No | No | ^m^ | ^m^ | Yes | ^m^ | ^m^ |
|  | Viral load | 1,335 | ^†^ | ^†^ | ^†^ | ^m^ | ^†^ | ^†^ | ^†^ | ^m^ |
|  | CD4 count | 316 | ^†^ | 236 | ^†^ | ^m^ | ^†^ | 354 | ^†^ | ^m^ |

* Reported ART use at exit interview; did not report ART use at any other visits.

^†^ Not measured by protocol

^m^ = Missing

ND = not detectable
