# Supplementary material for: Higher prevalence of viral control in HIV-1-infected women in serodiscordant relationships
Source: PLoS One. 2018 Dec 5;13(12):e0208401. doi: 10.1371/journal.pone.0208401 (PMC6281234; doi:10.1371/journal.pone.0208401)
Supplement: S3 Table — (DOCX) [file pone.0208401.s006.docx]

|  | *N* | FDC  *n* (%) |  | *N* ^a^ | FSW  *n* (%) ^a^ | Relative risk  (95% confidence interval) ^b^ | *p* |
| --- | --- | --- | --- | --- | --- | --- | --- |
| At baseline visit |  |  |  |  |  |  |  |
| Viral load < 150 copies/mL | 289 | 21 (7.3) |  | 220 | 3.1 (1.4) | 5.33 (2.66, 15.99) | < 0.001 |
| Viral load < 1,000 copies/mL | 289 | 31 (10.7) |  | 220 | 8.0 (3.6) | 2.95 (2.15, 4.72) | < 0.001 |
| Viral load < 2,000 copies/mL | 288 | 40 (13.9) |  | 220 | 12.2 (5.5) | 2.55 (1.91, 3.40) | < 0.001 |
| Durable viral control ^c^ |  |  |  |  |  |  |  |
| Viral load < 150 copies/mL | 208 | 16 (7.7) |  | 72.2 | 0.2 (0.3) | 7.60 (4.92, 8.90) | 0.002 |
| Viral load < 1,000 copies/mL | 208 | 25 (12.0) |  | 72.2 | 2.0 (2.8) | 4.39 (2.04, 12.31) | 0.006 |
| Viral load < 2,000 copies/mL | 208 | 29 (13.9) |  | 72.2 | 2.8 (3.9) | 3.49 (1.84, 11.73) | 0.011 |

^a^ Values are means of 5,000 bootstrapped datasets.

^b^ Values are median and 2.5^th^ and 97.5^th^ percentiles of distribution of 5,000 bootstrapped datasets.

^c^ Defined as viral control at baseline and one follow-up visit. Calculations include all women.

FDC = females in discordant couples; FSW = female sex workers
